# Genomic and chemical analyses of 713 marine biofilm-derived bacterial strains

**DOI:** 10.1128/aem.02593-25

**Published:** 2026-03-04

**Authors:** Jie Lu, Shen Fan, Jiaoxia Shi, Xiaoli Hu, Wei Ding, Jiejie Hao, Weipeng Zhang

**Affiliations:** 1 MOE Key Laboratory of Evolution and Marine Biodiversity and Institute of Evolution and Marine Biodiversity, Ocean University of China 535359 , Qingdao, China; 2 MOE Key Laboratory of Marine Genetics and Breeding and College of Marine Life Sciences, Ocean University of China 506915 , Qingdao, China; 3 MOE Key Laboratory of Marine Drugs and School of Medicine and Pharmacy, Ocean University of China 506918 , Qingdao, China; University of Delaware, Lewes, Delaware, USA

**Keywords:** marine biofilm, biosynthetic gene clusters

## Abstract

Marine biofilms are known as a reservoir of bacterial specialized metabolites, but the majority of these metabolites remain unexplored because most biofilm-associated bacteria have not yet been cultivated or genomically characterized. In a recent study, we isolated and cultivated 713 bacterial strains from marine biofilms and generated their nearly complete genomes. Here, we conduct a systematic analysis of biosynthetic gene clusters (BGCs) contained in these bacterial genomes. A total of 3,146 BGCs are predicted and organized into 2,176 mostly new gene cluster families (GCFs), in comparison with the GCFs in the Minimum Information about a Biosynthetic Gene cluster database, and those from genomes of global seawater bacteria. In particular, certain less-studied microorganisms, such as members of the *Roseobacteriaceae* family, possess a number of novel BGCs. Moreover, through bacterial antagonistic tests, 50 of the 713 strains inhibit the growth of at least one tested pathogenic bacterial strain. Furthermore, metabolomics followed by molecular networking reveals previously uncharacterized antimicrobial activities associated with known secondary metabolites, represented by the polycyclic tetramate macrolactam alteramide A.

**IMPORTANCE:**

Marine microorganisms are important sources of natural products, yet quite a few studies have systematically explored the production of active molecules by marine biofilm-associated bacteria. In the present study, we analyzed nearly complete genomes of 713 strains isolated from marine biofilms to assess their biosynthetic potential. We further conducted experiments to discover compounds with a strong inhibitory effect against pathogenic bacterial strains. This work has laid the groundwork for further prospecting marine biofilm-associated bacterial strains for antibacterial agents.

## INTRODUCTION

There is increasing evidence to show that marine microorganisms are important sources of natural products. These products have a range of bioactivities, such as antimicrobial, antitumor, and immunosuppressive, and thus are extensively utilized in medicine, aquaculture, and the cosmetic industry (1–3). In particular, antimicrobial molecules have been discovered in certain marine bacteria, such as tropodithietic acid, α-pyrones, and thiomarinol (4–6). These compounds display potent inhibitory effects on the growth of diverse human pathogenic strains, such as *Escherichia coli* and *Staphylococcus aureus* (4–6).

In marine environments, microorganisms exist in two major lifestyles: as free-living organisms or in association with a biofilm. While the biosynthetic potential of microorganisms living in seawater has been systematically explored (7, 8), quite a few studies have focused on the production of active molecules by marine biofilm bacteria. Biofilm formation can be tracked to the fossil record of about 3.25 billion years ago and is common among diverse microbial lineages (9, 10). Our recent studies on marine biofilms suggest that they are structurally complex and encompass hugely diverse taxonomic compositions, functions, and metabolisms (11–14). In particular, analysis of 479 metagenome-assembled genomes (MAGs) from biofilms developed in global marine environments has revealed a number of novel biosynthetic gene clusters (BGCs) (11). However, the paucity of cultured bacterial strains has long hindered the exploitation of useful metabolites from marine biofilms, because no experimental verification could be performed to verify the activity of predicted compounds.

In a recent study (15), we isolated and cultivated 713 non-redundant strains from biofilms on microplastics (*n* = 335) and stone surfaces (*n* = 378) and obtained their high-quality genomes. In the present study, to evaluate the biosynthetic potential of marine biofilm bacteria, we profiled the BGCs in the genomes of the 713 strains and further compared them with BGCs documented in a public database, as well as those encoded in genomes of 934 global seawater bacterial strains. Moreover, to show the bioactivity of the metabolites produced by the marine biofilm bacteria, we performed strain-to-strain inhibitory experiments against various human pathogens. Furthermore, through metabolomics, cheminformatics, and bioassay-guided purification, we uncovered previously uncharacterized antibacterial activities associated with known secondary metabolites.

## RESULTS

### Biosynthetic gene profiling of the marine biofilm-derived bacteria

The 713 strains were isolated from biofilms on microplastics (335 strains) and stones (378 strains) immersed in coastal zones, in our recent project focusing on bacterial isolation from marine biofilms (15). They are non-redundant strains with high-quality (average nucleotide identity [ANI] >99.9%, completeness >95%, and contamination <5%) genomes. Taxonomic annotation of the 713 marine biofilm-derived strains was then performed on the genomes using the Genome Taxonomy Database (GTDB) (16). At the phylum level (class level for Proteobacteria), the strains were assigned to Alphaproteobacteria, Gammaproteobacteria, Bacteroidota, Firmicutes, and Actinobacteriota (Fig. S1). At the genus level, *Qipengyuania*, *Psychrobacter*, and *Pseudoalteromonas* were overrepresented (Fig. S1). Other information, including taxonomic classification at different levels, genome size, genome completeness, data accession, and so on, is given in Table S1.

**Fig 1:**
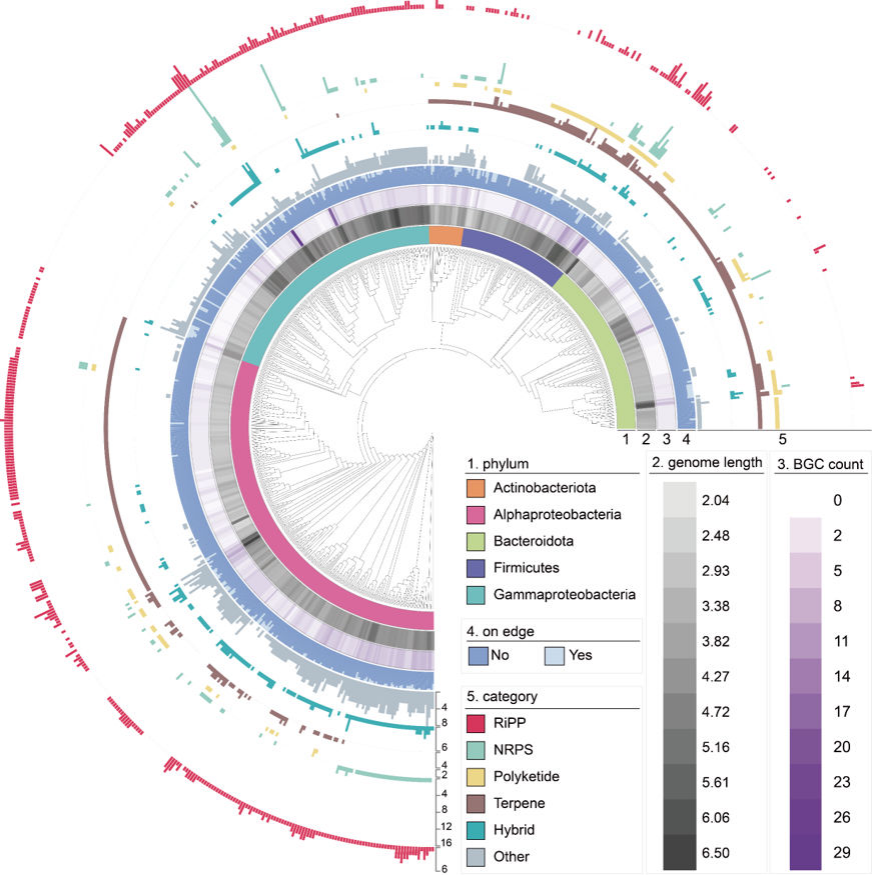
Distribution pattern of biosynthetic gene clusters (BGCs) across the species tree of the 713 strains. The concentric layers represent (inner to outer layers): phylum level affiliation, genome length, number of BGCs per genome, BGC completeness (on edge or not), and the category of BGCs.

To explore the biosynthetic potential of the marine biofilm-derived strains, antiSMASH (17) searching was conducted. A total of 3,146 BGCs were identified from the 713 genomes, with a median value of 4.41 BGCs per genome (the full list is given in Table S2). Less than 10% of the BGCs were on edges of contigs (Fig. 1; Table S2), suggesting that the high quality of genomes has largely facilitated BGC discovery. These BGCs were classified into six categories, with ribosomally synthesized and post-translationally modified peptide (RiPP) and terpene being the most abundant (21.3% and 17.3%, respectively) clusters (Fig. 1; Table S2). At the phylum level, RiPPs were prevalent among Alphaproteobacteria, while terpenes were prevalent among Bacteroidota and Firmicutes (Fig. 1).

**Fig 2:**
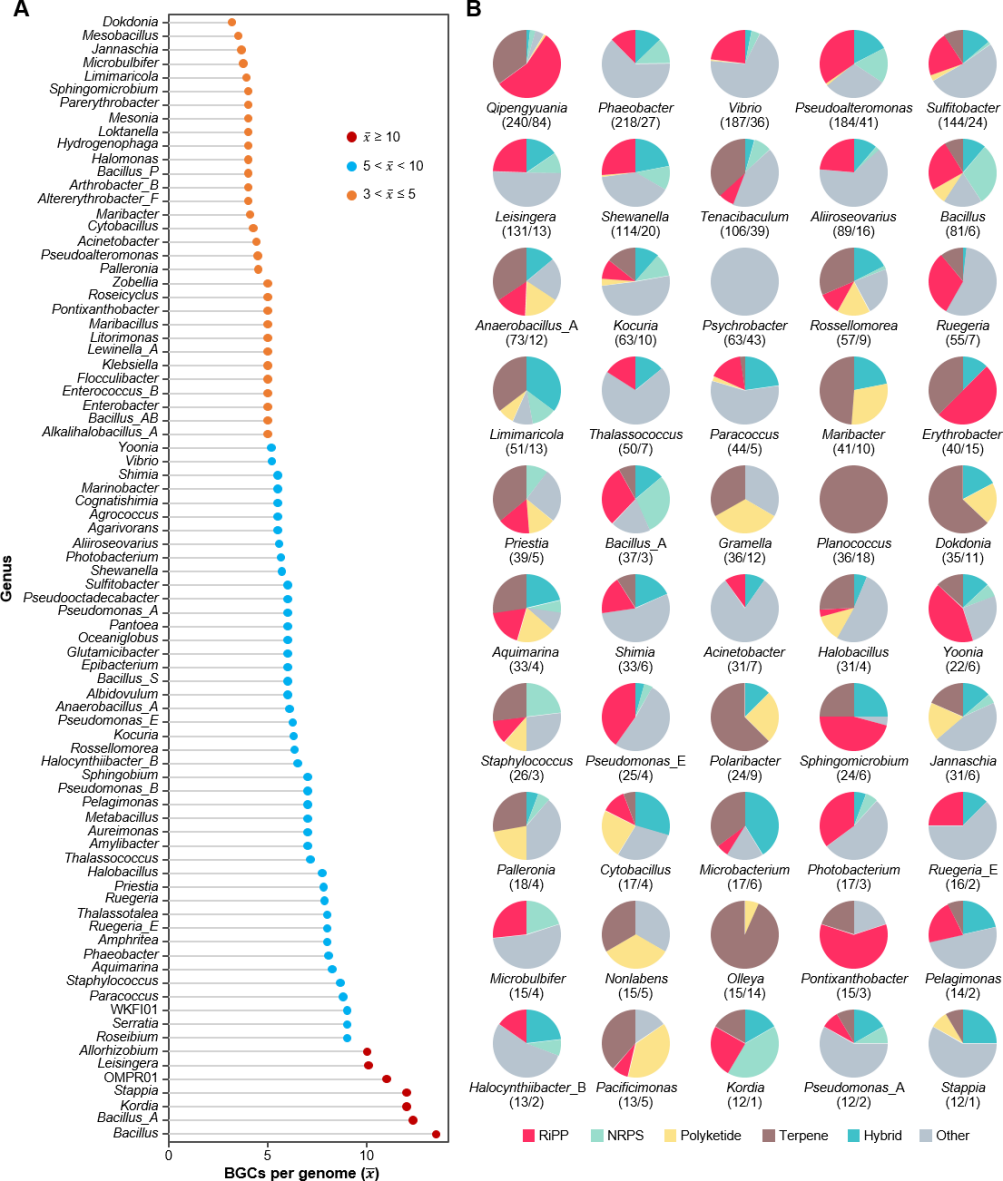
Density and distribution of BGCs at the genus level. (**A**) Density of BGCs at the genus level across biofilm-derived genomes ($\overset{-}{x}$ > 3). (**B**) Distribution of different types of BGCs in the top 50 genera with the highest BGCs content. Numbers in the brackets represent the ratio between BGCs and genomes.

At the genus level and in terms of the number of BGCs per genome, *Bacillus* was the most potentially high-yielding genus, with 13.5 BGCs/genome identified (Fig. 2A). The genera *Bacillus*_*A*, *Kordia*, and *Stappia* also encoded considerable numbers of BGCs per genome (Fig. 2A). Many of the potentially productive genera, such as *Leisingera*, *Phaeobacter*, and *Ruegeria_E*, were found to be affiliated with the family Roseobacteraceae, a typical marine bacterial group widely distributed in diverse oceanic niches (18–20). The BGC category distribution among the genera was visualized, with different genera displaying different biosynthetic potentials (Fig. 2B). For example, *Qipengyuania*, *Erythrobacter*, *Pseudomonas_E*, and *Pontixanthobacter* tend to biosynthesize RiPPs over other types of compounds; *Tenacibaculum*, *Maribacter*, *Planococcus*, *Dokdonia*, *Polaribacter*, and *Olleya* tend to biosynthesize terpenes over other types of compounds, whereas the major products of *Bacillus* and *Kordia* were likely to be nonribosomal peptides (NRPs; Fig. 2B).

### Novelty and specificity of the marine biofilm-derived gene cluster families

**Fig 3:**
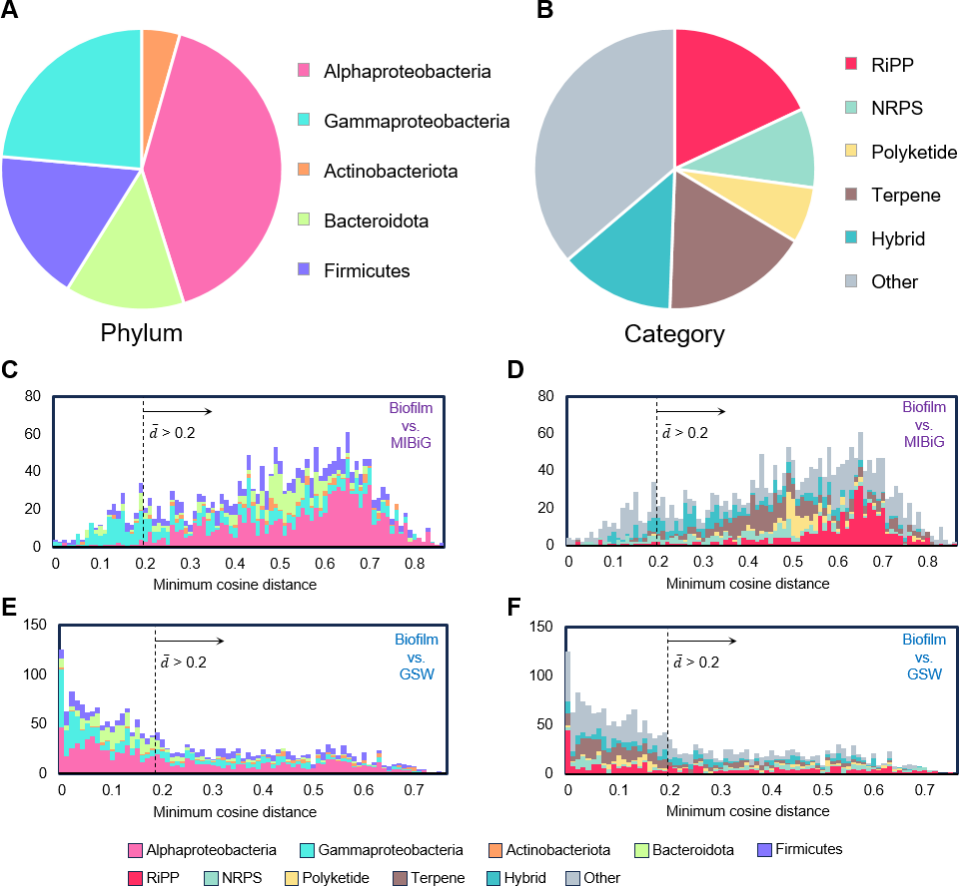
Novelty and specificity of gene cluster families (GCFs) derived from the marine biofilm strains. (**A**) Phylum-level distributions of the 2,176 GCFs. (**B**) Proportion of 2,176 GCFs across BGC categories. (**C and D**) Comparison of the biofilm-derived GCFs with those in the Minimum Information about a Biosynthetic Gene cluster (MIBiG) database. (**E and F**) Comparison of the biofilm-derived BGCs to the global seawater (GSW) BGCs. In panels **C–F**, the x-axis represents the mean minimum cosine distance ($\overset{-}{d}$) of each GCF against the MIBiG or GSW databases, with results from each database colored by phylum level classification and BGC category, respectively.

To evaluate their novelty, the biofilm-derived BGCs were classified into gene cluster families (GCFs), and then representative BGCs for the GCFs (one BGC for one GCF) were compared with those in the Minimum Information about a Biosynthetic Gene cluster (MIBiG) (21) database and those from the global marine bacteria. With a cosine distance threshold of 0.2, the biofilm-derived BGCs were classified into 2,176 non-redundant GCFs (the full list is given in Table S2). Consistent with the BGC profiling, Alphaproteobacteria contributed the largest proportion of GCFs, followed by Gammaproteobacteria and Firmicutes (Fig. 3A). In terms of category, most of the GCFs belonged to RiPPs, followed by terpenes and hybrids (Fig. 3B).

Comparison with the MIBiG database revealed 1,906 (87.6%) GCFs with a mean minimum cosine distance ($\overset{-}{d}$) over 0.2 to those in the MIBiG database (Fig. 3C and D). The novel GCFs were predominantly observed in Alphaproteobacteria (Fig. 3C). In terms of BGC category, the novel GCFs were majorly classified as RiPPs, and a considerable proportion of GCFs could not be functionally classified (named as others; Fig. 3D). Moreover, the marine biofilm BGCs were compared with those from the global seawater bacteria documented in a previous study (8, 22). These seawater-derived bacteria comprised 934 high-quality (completeness >95% and contamination <5%) genomes of bacterial isolates or single cells from a global ocean scale. As a result, 948 (43.6%) of the GCFs had a mean minimum cosine distance over 0.2 (Fig. 3E and F). Alphaproteobacteria recruited 40.8% of the novel GCFs (Fig. 3E), which majorly belonged to RiPPs (Fig. 3F). In addition, comparison with BGCs from previously known marine biofilm-associated bacteria showed that 814 (37.4%) of the GCFs had a mean minimum cosine distance greater than 0.2, further highlighting the substantial biosynthetic divergence of biofilm-derived strains (Fig. S2). Venn analysis of the common and specific GCFs between marine biofilms, global seawater, and MIBiG revealed only 2 common GCFs, whereas 2,033 GCFs were specific to marine biofilms (Fig. S3). Collectively, the above analyses implied high novelty and niche specificity of the biofilm-derived GCFs.

### Antimicrobial spectrum of the marine biofilm strain-derived products

Given the high cell density in marine biofilms, the bacteria may employ chemical molecules as their weapons to compete with each other (23). Thus, a large number of the marine biofilm bacteria-derived products are expected to be antimicrobial molecules. This motivated us to perform a screening on all the 713 marine biofilm strains with respect to antimicrobial activity. Eleven pathogenic bacterial strains were employed as indicators, including two gram-positive bacteria: *Staphylococcus aureus* ATCC 12600 and *Bacillus subtilis* ATCC 23857, and nine gram-negative bacteria: *Escherichia coli* ATCC 11775, *Acinetobacter baumannii* ATCC 19606, *Klebsiella pneumoniae* ATCC 17978, *Pseudomonas aeruginosa* PA01, *Cronobacter sakazakii* ATCC 29544, *Salmonella bongori* ATCC 43975, *Yersinia pseudotuberculosis* BNCC336701, *Vibrio parahaemolyticus* EMS, and *Vibrio alginolyticus* xv22. All these strains are human pathogenic or opportunistic strains, and several of them are multi-drug resistant strains (the information of these strains is given in Table S3).

**Fig 4:**
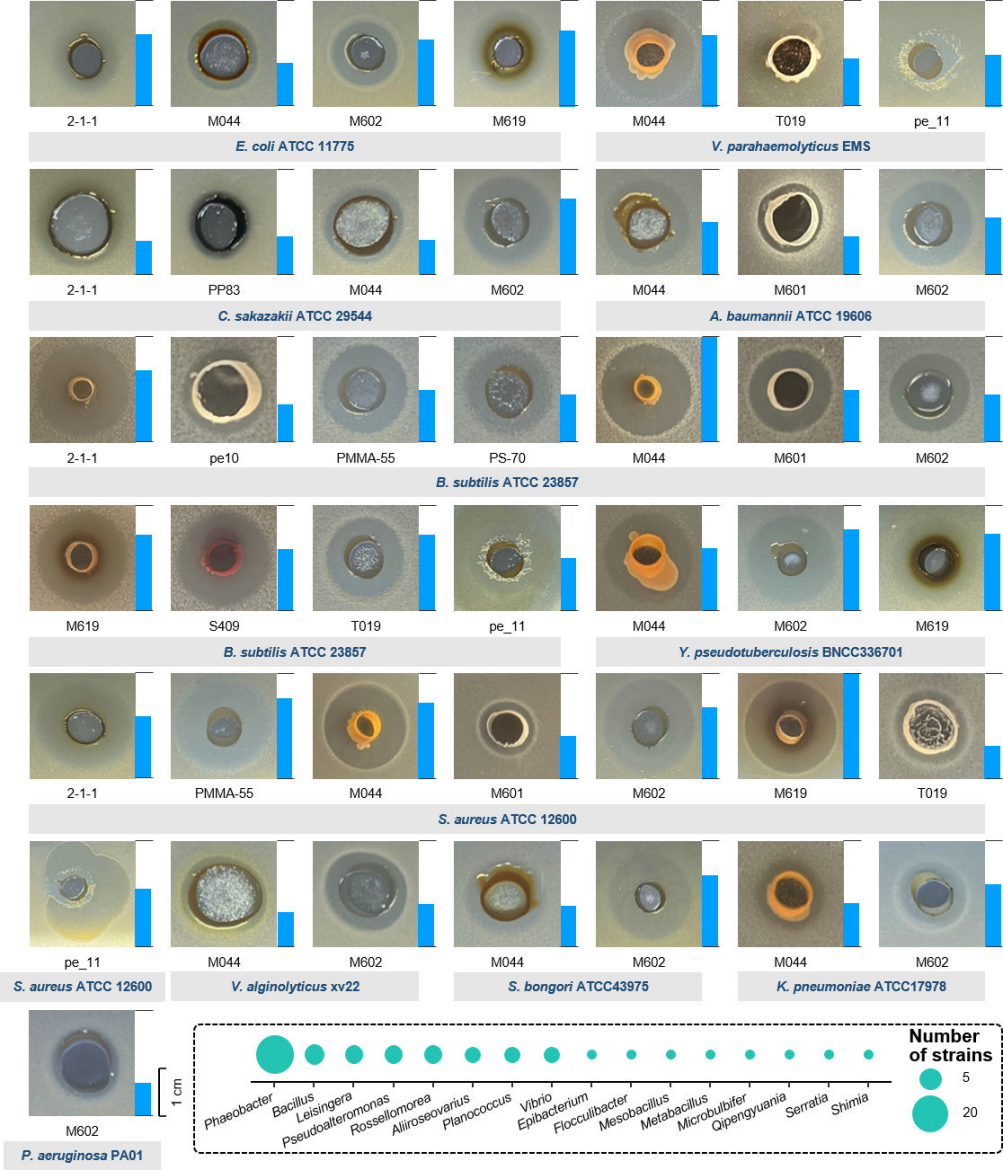
Antimicrobial activity of 12 representative strains against pathogenic bacteria. The pathogenic bacteria used for screening include *Staphylococcus aureus* ATCC 12600 and *Bacillus subtilis* ATCC 23857, *Escherichia coli* ATCC 11775, *Acinetobacter baumannii* ATCC 19606, *Klebsiella pneumoniae* ATCC 17978, *Pseudomonas aeruginosa* PA01, *Cronobacter sakazakii* ATCC 29544 *Salmonella bongori* ATCC 43975, *Yersinia pseudotuberculosis* BNCC 336701, *Vibrio parahaemolyticus* EMS, and *Vibrio alginolyticus* xv22. The bar on the right of an inhibition zone image indicates the diameter of the inhibition zone. The box in the lower-right corner shows genus-level affiliations of all 50 strains exhibiting antimicrobial activity.

Inhibition activity was examined by observing the inhibition zones after co-culturing on agar plates. The indicator strains were mixed with the agar, and the marine biofilm strains were seeded at the center of the plate using a gel punch. Of the 713 marine biofilm strains included in the experiment, 50 displayed antimicrobial activity against at least one pathogenic bacterial strain. The full list of the strains with inhibitory activity is given in Table S4. Many marine biofilm strains displayed inhibitory activities against *S. aureus* ATCC 12600, and strains with inhibitory activities against *B. subtilis* ATCC 23857, *E. coli* ATCC 11775, and *C. sakazakii* ATCC 29544 were also identified (Table S4). These bioactive strains were majorly from the genera *Phaeobacter*, *Bacillus*, *Leisingera*, and *Pseudoalteromonas*, and representative photos of the inhibitory results are shown (Fig. 4). To elaborate, the marine biofilm strain *Pseudoalteromonas elyakovii* M044 strongly inhibited *B. subtilis* ATCC 23857 and *S. aureus* ATCC 12600 (Fig. 4). The marine biofilm strain *Flocculibacter collagenilyticus* M601 displayed broad-spectrum antimicrobial activity against gram-positive and gram-negative pathogenic strains (Fig. 4). *Leisingera aquaemixtae* M602 displayed extensive antibacterial activity and was the only strain that inhibited the growth of *P. aeruginosa* PA01 (Fig. 4). Notably, several of the inhibitory zones reached a diameter of 2 cm, such as *P. elyakovii* M044 against *V. parahaemolyticus* EMS and *Phaeobacter inhibens* M619 against *S. aureus* ATCC 12600 (Fig. 4), suggesting potent inhibitory effect and probably diffusion of the antimicrobial molecules.

### Discovery of antimicrobials through mining of the marine biofilm strain products

To explore the compound produced by the marine biofilm-derived strains that displayed antimicrobial activities, metabolomics was conducted on 12 representative strains, each strain from a distinct genus. Their products, when cultured on agar media, were collected individually and subjected to liquid chromatography–tandem mass spectrometry (LC–MS/MS). The MS data were processed using the Global Natural Product Social Molecular Networking (GNPS-MN) (24) workflow. Following manual curation to remove subnetworks that were also present in the blank sample (medium only), the final molecular network comprised 2,655 nodes, including 1,689 singletons and 966 nodes interacting with at least one another node. The full list of the annotated metabolites was summarized in Table S5. These nodes were organized into 180 molecular families, each containing two or more nodes (Fig. S4). Within these 180 molecular families, 71 nodes were dereplicated as known metabolites using Dereplicator+ (score ≥ 12) (25), and 90 known metabolites were annotated (Table S5). Further analysis of the annotated metabolites revealed secondary metabolites with antibacterial properties. For example, the strain *P. elyakovii* M044 was suggested to produce the alteramide A and dibromoalterochromide A; the strain *Serratia marcescens* S409 was suggested to produce prodigiosin; and the strain *Bacillus altitudinis* PMMA-55 was suggested to produce surfactin A (Fig. S4).

**Fig 5:**
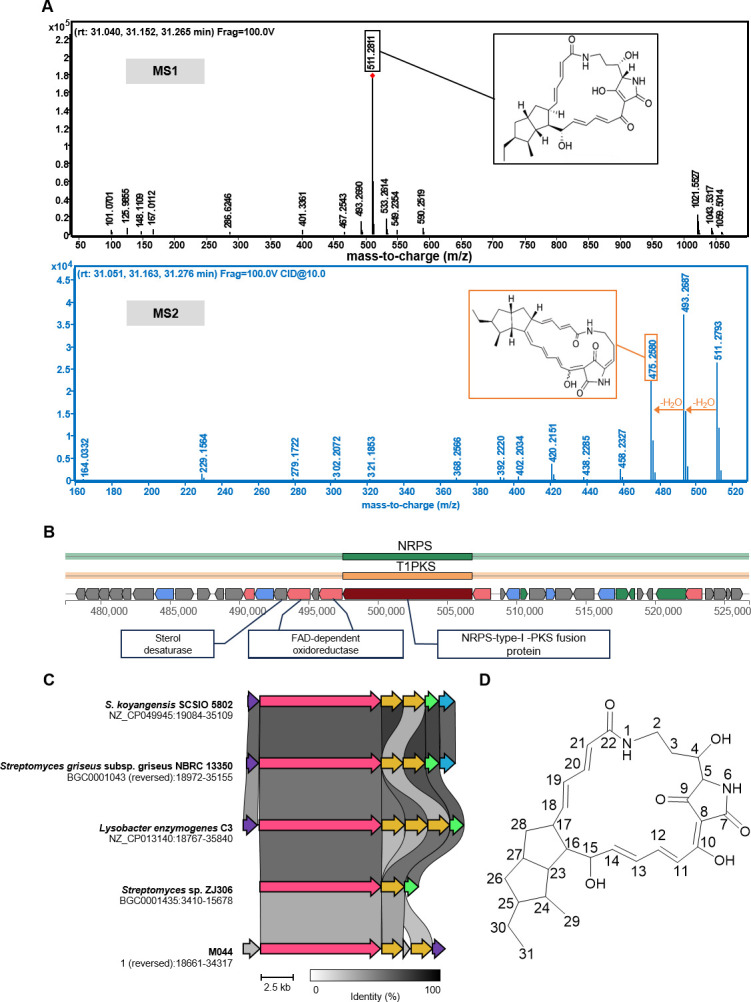
Identification of alteramide A produced by *Pseudoalteromonas elyakovii* M044. (**A**) MS1 and MS2 spectra of alteramide A in M044 crude extract. (**B**) The putative biosynthetic gene cluster responsible for the biosynthesis of alteramide A. (**C**) Comparative analysis of alteramide A BGC with other reported polycyclic tetramate macrolactam BGCs. (**D**) Chemical structure of alteramide A isolated from strain M044.

To confirm the production of the aforementioned molecules, MS data were visualized. For the data of *P. elyakovii* M044, the ion at *m/z* 511.2811 corresponded to the protonated molecular species [M + H]^+^ of alteramide A, while the ion at *m/z* 533.2614 corresponded to its alkali metal adduct [M + Na]^+^ (Fig. 5A). Moreover, during MS/MS analysis, two additional ion fragments (*m/z* = 493.2687, 475.2580) of alteramide A were detected, indicating that the molecule undergoes dehydration [M + H − H_2_O]^+^ (Fig. 5A). The corresponding BGC was identified in the genome of *P. elyakovii* M044, with the presence of key enzymes sterol desaturase, FAD-dependent oxidoreductase, and NRP-type-I-PKS fusion protein (Fig. 5B). This BGC exhibited high similarity to previously reported polycyclic tetramate macrolactam gene clusters (Fig. 5C) (26–29). *P. elyakovii* M044 could also produce the yellow pigment dibromoalterochromide A, probably using the NRP synthetase, fatty acid synthase, and flavin-dependent halogenase as key enzymes (Fig. S5). In addition, the MS/MS result suggested that strain *F. collagenilyticus* M601 also produced alteramide A, with the detection of ions at *m/z* 511.2783, 493.2642, and 475.2588 (Fig. S6). However, no respective BGC was annotated in its genome, probably due to the low similarity of its BGC with that documented in the antiSMASH database. Besides, many strains, such as *Vibrio proteolyticus* pe_11, *L. aquaemixtae* M602, and *Aliiroseovarius crassostreae* PP83 generated a substantial number of metabolites that remain unannotated (Table S5).

### Antibacterial activity of alteramide A

To further characterize the antibacterial component, the crude extract of *P. elyakovii* M044 was purified, yielding the compound corresponding to *m/z* 511.2811 as a yellow solid. Structural elucidation by NMR spectroscopy identified it as the polycyclic tetramate macrolactam alteramide A (Fig. 5D; Fig. S8 to S12; Table S6). Disk diffusion assays demonstrated that alteramide A effectively inhibited the growth of *S. aureus* ATCC 12600 and *B. subtilis* ATCC 23857 (Fig. S7). Further quantification of its potency revealed a strong antibacterial activity, with MIC values as low as 8 μg/mL against both strains. Notably, antibacterial activity has not been previously reported for alteramide A.

## DISCUSSION

In the current study, through combining genomic analysis, antimicrobial experiments, and metabolomic analysis, we systematically profiled the biosynthetic potential of culturable marine biofilm bacteria and identified previously uncharacterized antibacterial activities of metabolites.

In a previous study (11), we have explored the biosynthetic profiles of marine biofilm-associated bacteria through metagenomics, which revealed a number of novel biosynthetic gene clusters. Here, by focusing on pure-cultured strains, our findings have further illuminated the chemical biosynthetic space of marine biofilm-associated bacteria, and significant progress has been made. First, the genomes of cultured strains are much more complete than MAGs. Although metagenomic data can provide bacterial genomic information in a culture-independent way, the fragmentation and incompleteness of the MAGs may pose considerable challenges for comprehensive analysis, considering that many BGCs are long gene clusters (30, 31). For the isolated strains, less than 10% of the BGCs were on edges of contigs, suggesting that most of the recovered BGCs are complete. Second, pure cultured strains allow for laboratory experiments, such as the antimicrobial assay, as well as the chemical extraction and LC-MS/MS detection. These experiments are pivotal for the discovery of novel compounds and the identification of their chemical structures (32, 33).

Within the genomic analyses, comparison with the MIBiG database highlights the novelty of BGCs from the marine biofilm-derived bacteria. Following the same scenario, nearly half of marine biofilm-derived BGCs exhibit unique characteristics when compared with BGCs from the reference seawater bacteria, although the latter were collected from global ocean environments (8). This result implies that the biosynthesis of chemical molecules is likely to be influenced by niche differentiation. For example, the marine biofilm-derived *A. crassostreae* encodes unique type-IV lanthipeptide BGCs and butyrolactone gene cluster that are absent from its seawater-derived counterparts, and it also harbors multiple hybrid BGCs with complex modular architectures. Inter-strain variability exists within several genera represented in marine biofilms, including *Bacillus* (*B. altitudinis*), *Aliiroseovarius* (*A. crassostreae*), *Leisingera* (*L. caerulea*), and *Phaeobacter* (*P. inhibens*). Consequently, future bioprospecting efforts should prioritize these genera, as strain-level isolation from their biofilms is a promising avenue for uncovering untapped chemical diversity. In line with this notion, environmental structures could be affected by niche differentiation and then pose impacts on microbial composition and secondary metabolism (34). Moreover, while marine *Bacillus* and *Pseudomonas* are well-known producers of useful compounds (35, 36), certain less-studied microorganisms, particularly those of the Roseobacteriaceae family (e.g., *Leisingera* and *Aliiroseovarius*), possess a number of novel BGCs. These findings underscore the potential of biofilms as a significant and unique source of natural products.

The antimicrobial experiments following genomic analyses reveal many strains with notable antibacterial effect. In particular, the marine biofilm-derived bacteria *P. elyakovii* M044, *F. collagenilyticus* M601, and *L. aquaemixtae* M602 inhibit many of the tested pathogens, including both gram-positive and gram-negative bacteria, implying the production of broad-spectrum antimicrobial molecules. Metabolomics using LC-MS/MS and chemoinformatics using GNPS-MN indicate that M044 can produce alteramide A and dibromoalterochromide A, and the strain M601 can also produce alteramide A. The compound alteramide A was previously found to be produced by *Alteromonas* sp. associated with marine sponge (37), *Pseudoalteromonas* sp. associated with marine coral (38), and *Streptomyces griseus* NBRC 13350 (27). While alteramide A produced by the coral-associated *Pseudoalteromonas* strain showed anti-fungal activity (38), no antimicrobial activity was previously reported for this compound. Bromoalterochromide A was found to be produced by a *Pseudoalteromonas* strain isolated as an epibiont of marine sponge (39). It was expected that this bacterium could also produce dibromoalterochromide A, but the activity was not investigated due to decomposition (39). Here, our findings indicate that alteramide A is a potent antimicrobial molecule with broad-spectrum activity, as confirmed by its low MIC values against the tested pathogens.

Altogether, our findings underscore the high potential of marine biofilm bacteria for the synthesis of natural products, many of which exhibit previously unreported antibacterial activity. Moreover, a considerable proportion of marine biofilm strains with potent antimicrobial activities have no annotated compounds, suggesting the production of novel antimicrobial molecules (e.g., certain antimicrobial peptides that cannot be recovered by antiSMASH). In addition, it should be noted that, although a number of RiPP BGCs were predicted in the genomes, respective compounds were not detected during the LC-MS/MS and GNPS-MN analysis. This inconsistency might be attributed to the silence of RiPP clusters and also warrants further exploration.

## MATERIALS AND METHODS

### Genome quality evaluation and basic analysis

The completeness and potential contamination of assembled genomes were assessed using CheckM2 (version 1.0.1) (40). High-quality genomes were defined as those that were over 95% complete and had less than 5% contamination. The contig N50 and genome size were then calculated using the software SeqKit (41), an ultrafast toolkit for FASTA/Q file manipulation. Taxonomic classification of the genomes was conducted using GTDB-Tk (version 2.3.2) (42), with the minimum alignment fraction to assign a particular genome to a species cluster being 50%. The whole-genome phylogenetic tree based on 120 bacterial universal marker gene set generated by GTDB-Tk was visualized using ITOL (version 6.9.1) (43).

The genomes of global seawater bacteria were downloaded from MarDB (version 1.6). Genomes matching the regular expression [C|c]ulture |[I|i]solate were selected. After filtering for the seawater environment, a total of 1,190 genomes were available. After removing genomes not associated with biofilm or surface-attached environments, a total of 2017 genomes of known marine biofilm bacteria were available. The quality of these genomes was further assessed using CheckM2; 934 global seawater bacteria genomes and 1,607 known marine biofilm bacteria genomes (completeness >95% and contamination <5%) were retained for comparison with marine biofilm bacteria.

### BGC prediction and analysis

BGCs were predicted from the genomes of marine biofilm bacteria, and global seawater bacteria were analyzed using antiSMASH (version 6.1.1, default parameters). The identified BGCs were categorized into six groups: “post-translationally modified peptide (RiPP),” “Nonribosomal peptides (NRP),” “Polyketide,” “Terpene,” “Hybrids,” and “Others.” Lanthipeptides were manually classified into the “RiPP.” Before GCF clustering, the BGCs predicted from genomes of marine biofilm bacteria and global seawater bacteria and those documented in the MIBiG 3.1 database were pooled together and subjected to feature (PFAM domains) extraction using the BiG-SLiCE tool (version 1.1.0) (44). These features were then utilized to compute pairwise cosine distances between all BGCs by scikit-learn (version 1.2.2) (45). Subsequently, the BGCs were clustered into GCFs with distance thresholds of 0.2 using AgglomerativeClustering (linkage = average) in scikit-learn. The GCF-GCF distance was defined as the minimum cosine distance between BGCs encompassed by the two GCFs. During comparison between BGCs from marine biofilms and MIBiG, the novelty of a given GCF in marine biofilms was indicated by its distance to the closely related GCF in MIBiG. Similarly, during comparison between marine biofilms and global seawater, the novelty of a given GCF in marine biofilms was indicated by its distance to the closely related GCF derived from genomes of global seawater bacteria. Putative BGCs were directly aligned to reference BGCs extracted from the downloaded genomes using Clinker (version 0.0.31) (46).

### Antimicrobial experiment

The antimicrobial activities of all cultured strains were tested using a radial diffusion assay (47). Nine gram-negative and two gram-positive bacterial strains were used as indicator strains. The 713 pure cultured strains from marine biofilms were cultured in liquid Marine Broth 2216E at 25°C without shaking for 72 h. The indicator strains were cultured overnight at 37°C in liquid Marine Broth 2216E. A volume of 10 μL culture of the indicator strains was supplemented to 20 mL not yet solidified medium with agar to prepare a uniform layer in a 90 mm diameter petri dish. The plates were left to dry for 1 h, and evenly spaced wells were made using a gel punch with a 5 mm diameter. Then, 50 µL of resuspended cultures of the marine biofilm strains were loaded into the wells in the agar plates and allowed to diffuse at 25°C. After 24 h, the sizes of the inhibitory zones were measured, and photos were taken.

### LC-MS/MS experiment and GNPS-MN analysis

The marine biofilm strains with antimicrobial activity were cultured on Marine Agar 2216E at 25°C for 7 days. The cells were scraped from the plates and extracted using 1.5 mL of methanol (high-performance liquid chromatography [HPLC] grade) in a benchtop tank sonicator for 20 min. Extracts were then centrifuged for 10 min at 12,000 rpm, and the supernatant was transferred into an Eppendorf tube, filtered through 0.22 µm nylon membrane filters, and subjected to UPLC-qTOF-MS. Blank samples were generated by extracting the cultivation medium.

Liquid chromatography separation took place on a Waters ACQUITY UPLC HSS T3 (2.1 × 100 mm × 1.8 uµm) housed in a column oven maintained at 35°C. A binary mobile phase gradient consisting of solvent A (acetonitrile) and solvent B (0.1% formic acid in water) was used, and an injection volume of 1 µL was applied. The gradient conditions were as follows: 0–40 min, 0%–70% mobile phase A and 100%–30% mobile phase B; 40–48 min, 70%–100% A and 30%–0%B; 48–51 min, 100% A and 0%B; 51–51.5 min, 100%–0% A and 0%–100%B; 51.5–56 min, 0% A and 100% B, with flow rate of 0.3 mL min^−1^. The chromatographic effluents were analyzed using a quadrupole time-of-flight (qTOF) high-definition mass spectrometer (Agilent 6530) in positive electrospray ionization mode. The instrument settings were as follows: mass range 100qTOF 3,200 m/z, ion spray voltage 4 kV, sheath gas temperature 350°C, sheath gas flow rate 11 L min^−1^, dry gas temperature 300°C, and dry gas flow rate 5 L min^−1^. For collision-induced dissociation, a collision energy gradient was applied at 10, 20, 40, and 60 eV, enabling the fragmentation of precursor ions and enhancing the identification of compounds.

All raw MS/MS data files were converted to 32-bit mzXML format using MSConvert software and subsequently uploaded to the GNPS platform (https://gnps.ucsd.edu) via WinSCP (https://winscp.net). The MN was then generated according to the online workflow with specific parameters: minimum cosine value of 0.70, minimum matching peak of 6, parent mass and fragmentation tolerance of 0.02 Da, and maximum connected component size of 100. In addition, the MGF file from MN was uploaded to the Dereplicator+ workflow in GNPS (http://gnps.ucsd.edu) with default parameters. Finally, the results were exported to Cytoscape (version 3.10.1) (48) software for visualization.

### Isolation and bioactivity characterization of antibacterial compounds from M044

*P. elyakovii* strain M044 was cultured on Marine Agar 2216E at 25°C for 7 days. The cells were scraped from the plates and extracted using methanol (HPLC grade) in a benchtop tank sonicator for 20 min. Extracts were then centrifuged for 10 min at 12,000 rpm, and the supernatant was transferred into an Eppendorf tube. The methanol in the extracts was evaporated to dryness using a vacuum centrifuge concentrator, and the residue was resuspended in fresh methanol using the benchtop ultrasonic bath for 2 min. Extracts were then centrifuged for 10 min at 12,000 rpm, and the supernatant was collected, filtered through 0.22 µm nylon membrane filters, and subjected to HPLC for the fractionation and purification of the target compound.

The disk diffusion method was used to investigate the effect of the antimicrobial activity of bacterial extracts on the tested pathogen strains. Twenty microliters of the compound solution was applied onto sterile 6 mm diameter filter paper disks, which were then placed on Müeller-Hinton agar plates previously inoculated with the test pathogenic strains. The plates were incubated at 37°C for 24 h, and the diameters of the inhibition zones were measured to assess antimicrobial activity.

### Determination of MIC for alteramide A

*S. aureus* ATCC 12600 and *B. subtilis* ATCC 23857 were cultured in cation-adjusted Mueller-Hinton broth (CAMHB) at 37°C to the logarithmic growth phase (OD600 = 0.5). Bacterial cultures were then diluted with fresh CAMHB to a density of 5 × 10^5^ CFU/mL. Antibacterial assays were performed in 96-well plates with a series of alteramide A concentrations (dissolved in DMSO) ranging from 2 to 64 μg/mL. Four experimental groups were set up for each strain: blank control: CAMHB alone, DMSO control: bacterial culture plus DMSO, experimental group: bacterial culture plus alteramide A solution, and solvent control: CAMHB plus DMSO. Three biological replicates were performed for each condition. Plates were incubated at 37°C for 24 h, and OD_600_ values were measured. The OD_600_ values of the blank control were subtracted from all experimental values to eliminate background. The MIC of alteramide A for each bacterium was defined as the lowest concentration at which no visible bacterial growth was observed.

### NMR measurements

The NMR spectra were recorded on an Agilent DD2 500 spectrometer (Agilent Technologies Inc., USA). Data were analyzed using the MestReNova software (version 15.0.0). For NMR acquisition on the 500 MHz, 10 mg of compounds dissolved in 600 μL of DMSO-*d*6 were used.

## Data Availability

The strains reported in this paper have been preserved in China General Microbiological Culture Collection Center (CGMCC, https://cgmcc.net/). All the strains can be freely accessed through the strain names listed in Table S1. The genome sequence data have been deposited in the Genome Warehouse in National Genomics Data Center, Beijing Institute of Genomics, Chinese Academy of Sciences, under the accession number PRJCA019328. Please see Table S1 for the accession number for each genome. Alternatively, the genomes can be downloaded from figshare with one click (https://figshare.com/s/decb10d44c2e8657cadf?file=49018204).

## SUPPLEMENTAL MATERIAL

Supplemental figures aem.02593-25-s0001.docx Figures S1 to S13.

Supplemental tables aem.02593-25-s0002.xlsx Tables S1 to S6.

ASM does not own the copyrights to Supplemental Material that may be linked to, or accessed through, an article. The authors have granted ASM a non-exclusive, world-wide license to publish the Supplemental Material files. Please contact the corresponding author directly for reuse.
