# Supplementary material for: Genomic and chemical analyses of 713 marine biofilm-derived bacterial strains
Source: Appl Environ Microbiol. 2026 Mar 4;92(4):e02593-25. doi: 10.1128/aem.02593-25 (PMC13101523; doi:10.1128/aem.02593-25)
Supplement: Supplemental figures — Figures S1 to S13. [file aem.02593-25-s0001.docx]

**Supplementary Figures for**

**Lu et al.**

**Genomic and chemical analysis of 713 marine biofilm-derived bacterial strains**


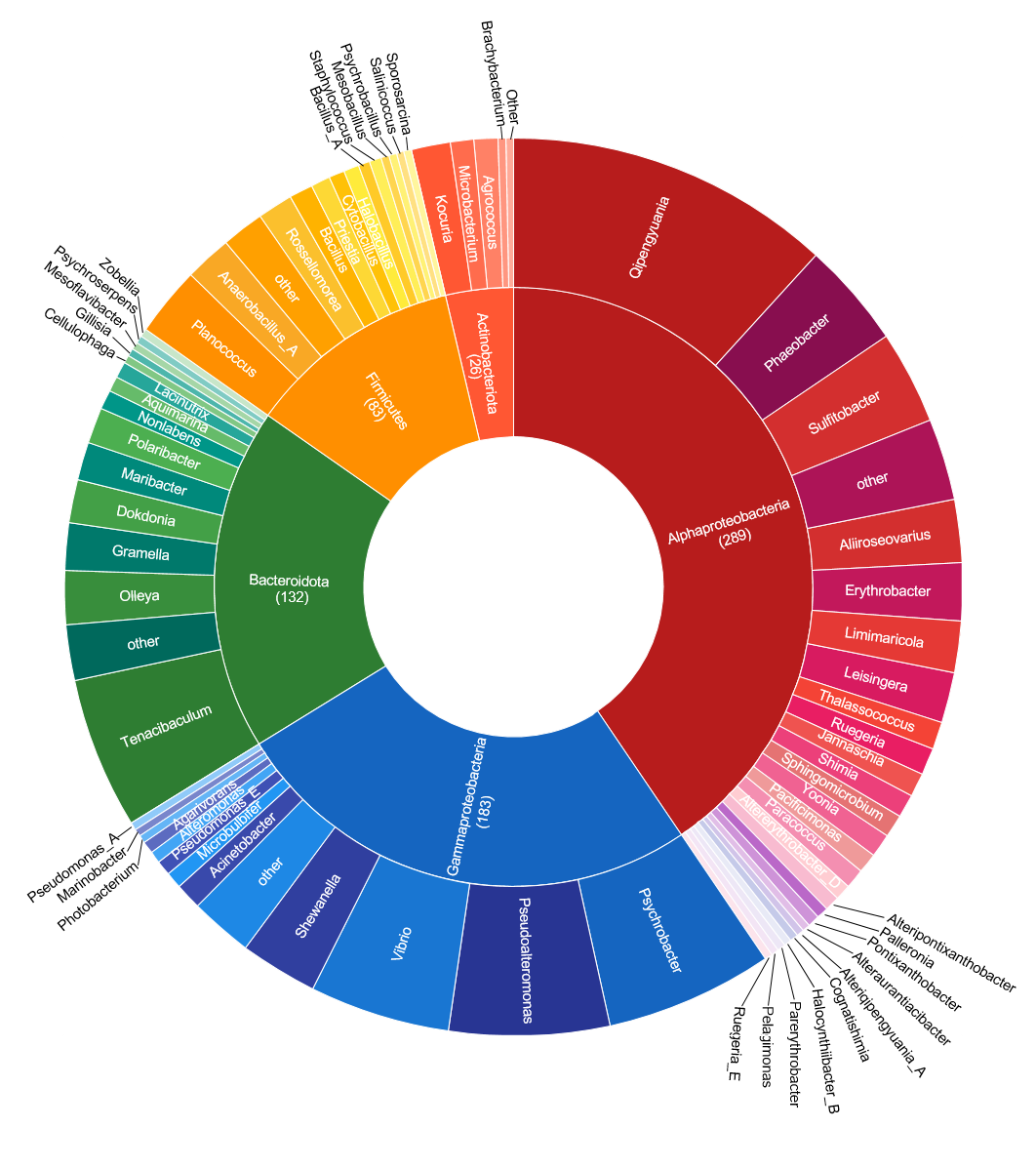


**Fig. S1 Distribution of the 713 biofilm-derived bacterial genomes across phylum- and genus-levels.**

The inner layer represents phylum-level classification, with the numbers in parentheses indicating the number of genomes, while the outer layer represents genus-level classification.


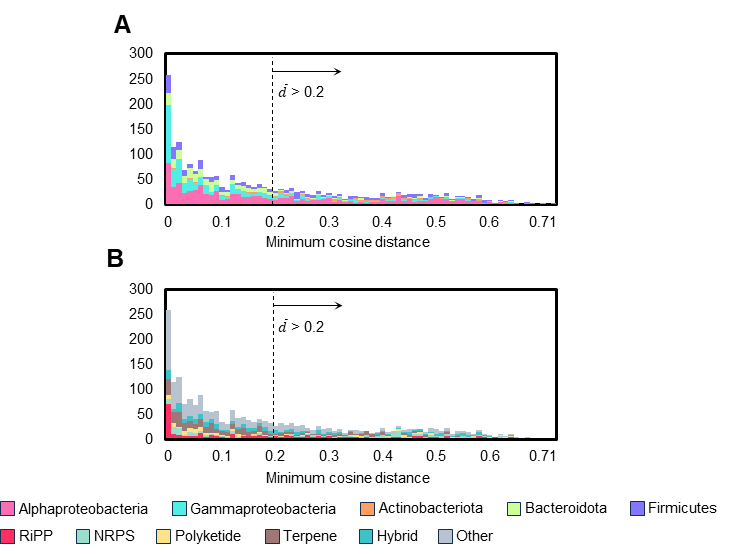


**Fig. S2 Comparison of biofilm-derived BGCs with those from previously reported marine biofilm-associated bacteria.**

In figures A and B, the x-axis represents the mean minimum cosine distance ($\bar{d}$) of each GCF against the reference biofilm BGC databases, with results from each database colored by phylum level classification and BGC category, respectively.


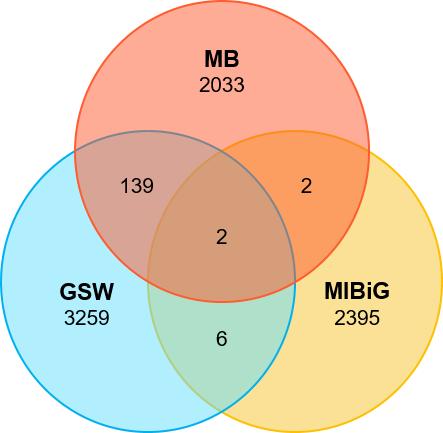


**Fig. S3 Venn analysis of the common and specific gene cluster families (GCFs) between marine biofilms (MB), global seawater (GSW), and the Minimum Information about a Biosynthetic Gene cluster (MIBiG) database.**

The GCFs were clutered using a cosine distances threshold of 0.2 between the BGCs.


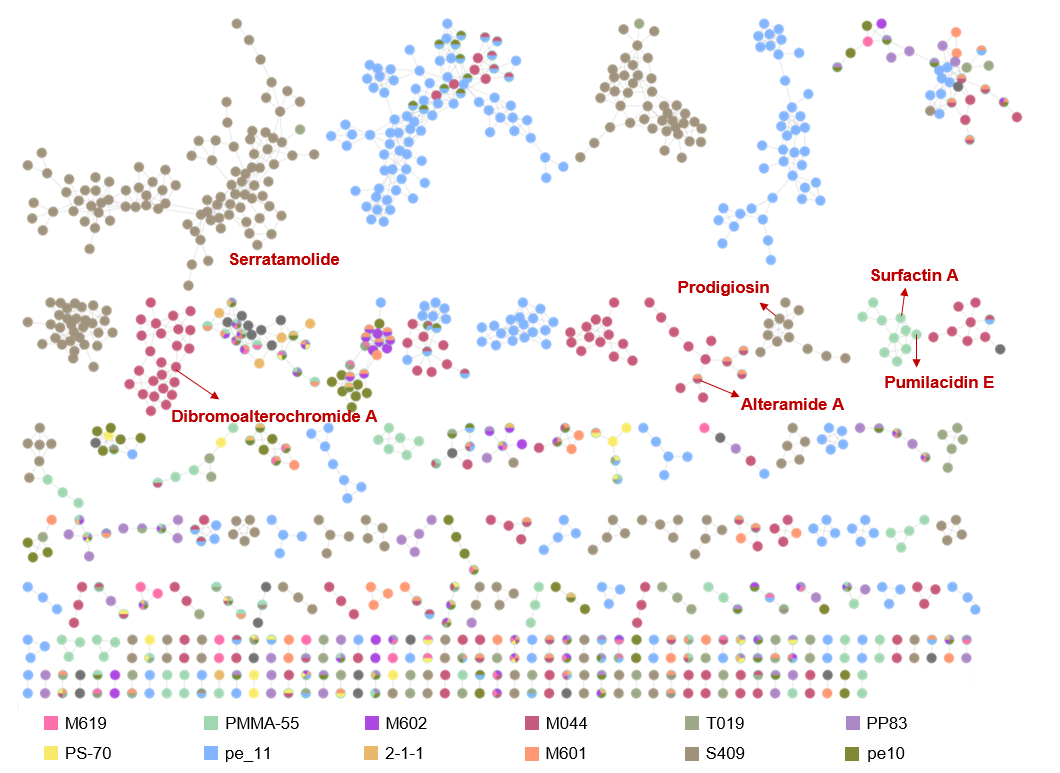


**Fig. S4 Molecular network of extracts of the 12 representative strains with strong antimicrobial activity.**

The node color reflects the producing strains responsible for the production of the metabolites.


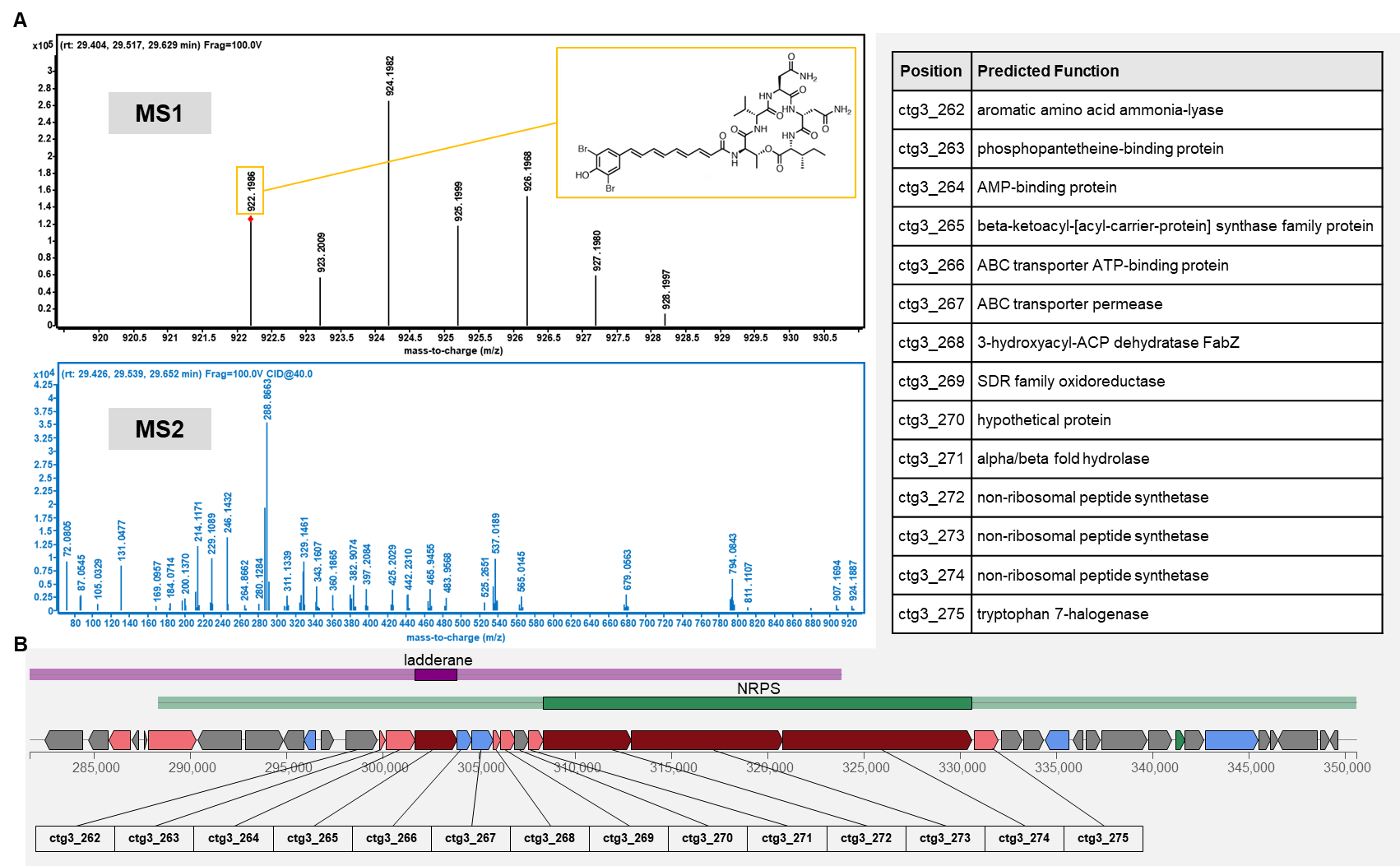


**Fig. S5 Identification of dibromoalterochromide A produced by *Pseudoalteromonas elyakovii* M044.**

(**A**) MS1 and MS2 spectra of dibromoalterochromide A in M044 crude extract. (**B**) The putative biosynthetic gene cluster responsible for the biosynthesis of dibromoalterochromide A.


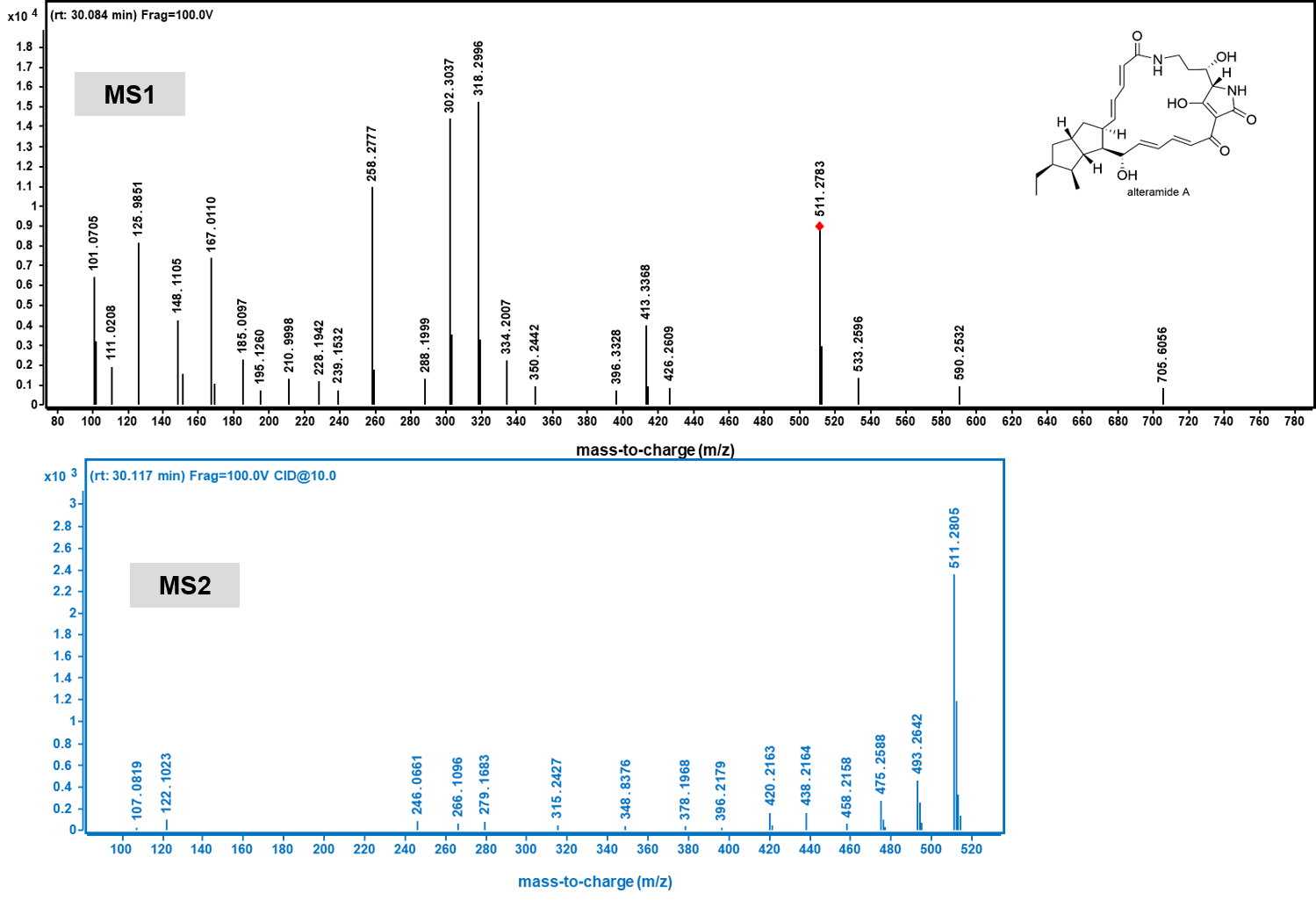


**Fig. S6 MS1 and MS2 spectra of alteramide A in *Flocculibacter collagenilyticus* M601 crude extract.**

**
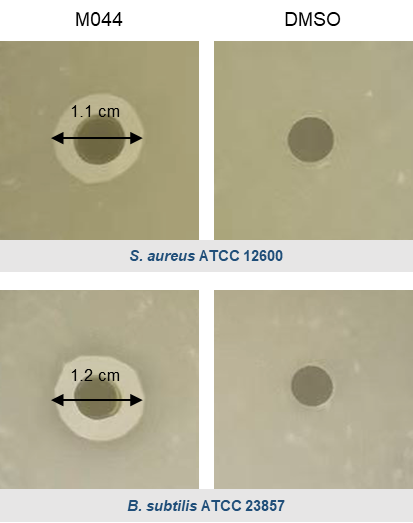
**

**Fig. S7 Antimicrobial activity of alteramide A against pathogenic bacteria.**

**Fig. S8 ^1^H NMR spectrum (500 MHz, DMSO-*d6*) of alteramide A.**

**Fig. S9 ^13^C NMR spectrum (500 MHz, DMSO-*d6*) of alteramide A.**

**Fig. S10 HSQC spectrum (500 MHz, DMSO-*d6*) of alteramide A.**

**Fig. S11 ^1^H-^1^H COSY spectrum (500 MHz, DMSO-*d6*) of alteramide A.**

**Fig. S12 HMBC spectrum (500 MHz, DMSO-*d6*) of alteramide A.**

**
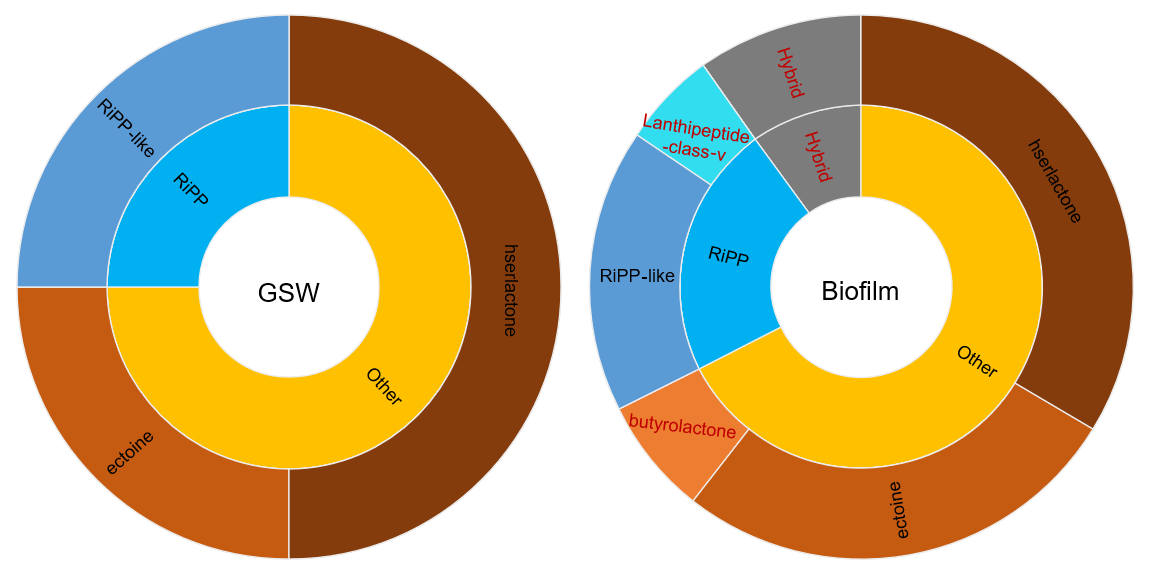
**

**Fig. S13 Comparison of BGC types encoded by *Aliiroseovarius crassostreae* from GSW and biofilms.**

The left panel shows the distribution of BGC categories (inner ring) and products (outer ring) in seawater-derived *A. crassostreae*, while the right panel represents the BGC categories (inner ring) and products (outer ring) in biofilm-derived *A. crassostreae*.
